# Monthly Summary

**Published:** 1883-01

**Authors:** 


### MONTHLY SUMMARY.

*Resolution of Alveolor Abscesses into Serous Cysts.*—Dr. Willoughby Weiss, L. D. S. England, says that E. P,—æt. 28, married, came to consult me, at the Western General Dispensary, respecting two swellings in the mouth.

The history she gave was that about three years ago she had an attack of erysipelas, and during the time suffered great pain with her teeth. She ultimately recovered from the erysipelas, but still continued to suffer with tooth-ache ; she did not, however, seek advice, and at the end of two or three weeks these swellings occurred, and had remained ever since. At this time she lost all pain, and only suffered a very little since when the weather was damp, which caused the tumor to slightly increase in size. At the early part of the disorder she had noticed a very nasty taste in her mouth, but this for some months had almost entirely disappeared.

Upon examination I found one tumor about the size of a walnut situated on the left side of the superior maxilla, in the region of the first molar, the roots of this tooth being in the jaw (as indeed were the roots of all the molars,) and somewhat loose. There was slight fluctuation, although not very marked, and no *craquement.* I removed the roots, expecting pus to flow from the opening, but instead, a clear thin glairy fluid escaped into the mouth, showing that the alveolar abscess, which it had undoubtedly been, had resolved into a serous cyst. The walls almost entirely collapsed, but I assisted the flow of the fluid by a little digital pressure and then syringed the cavity out with very dilute sulphate of zinc. The second swelling was on the left side of the inferior maxilla, in the region of the first bicuspid, the root remaining in the head, and with all the conditions presented in the upper jaw. I at once removed this root and a clear thin glairy fluid escaped, as in the other tumor. I treated this in the same way, pressing in the walls and syringed the cavity out with very dilute sulphate of zinc.

I then directed the patient to see me at the end of a week. By this time the swelling in the lower bed had entirely disappeared, but a probe could be passed a short way down where the root had been removed; in the upper, the alveolus was at the anterior part slightly distended. I crushed this with the fingers, and told the patient to come again in a week's time. Everything was now in a normal condition, the swellings having entirely disappeared, the opening for the roots having almost entirely closed up by granulations, so I discharged the patient. —*British Journal of Dental Science.*

---

*A New Remedy for Spasms.*—The *Med. Central Zeit.*, of July 15, 1882, publishes the result of experiments instituted by Dr. Schiffer, of Berlin, for the purpose of determining the therapeutic value of the *extract of Guachamaca*, a tree indigenous on the Apuche mountains. As a specific for all spastic conditions of the motor apparatus we so far possessed, in reality, one remedy only; curare, which, however, on account of its danger, and the uncertainty of its action, as well as of the remedy itself, has mostly been used for physiological experiments alone, and, perhaps, occasionally in tetanus.

Guachamaca extract, prepared from the bark of the Quebracho plant, which belongs to the same class as the Oleänder (Nerium ol. L.) and of which latter the skin between tree and bark is also poisonous, is a remedy which, while not so dangerous in its effects, is far more reliable and uniform in its action, and can besides easily be procured pure and genuine, and of always equal strength, as it is by no means rare.

Schiffer experimented with this drug, and made his observations in the clinic of Prof. Frerichs. He employed the remedy in solution, in the dose of ⅙ of a grain, and by the hypodermic method, in cases of tonic and clonic spasms of the muscular system, and always with a very good effect. He noted also that, administered internally, no matter in what solution, the drug was as little absorbed by the mucous membrane of the elimentary canal as curare is.

Should further trials with this remedy prove invariably as successful, or at least frequently so, as was the case in Schiffer's experiments, we would at last possess a reliable specific for all spasmodic affections of muscles and motor nerves, a drug, the physiological actions of which would be exactly opposite to that of strychnine and similar remedies.—*Med. and Surg. Reporter.*

---

*Hemorrhage after Teeth Extraction.*—Male, had eight teeth extracted nearly a week ago and is still suffering from hemorrhage.

An examination of the sockets of the teeth shows that the operation has been skillfully done. I can discover no fracture of the jaw, and no extensive ulceration of the gum. Ordinarily, bleeding from the socket of the tooth is arrested in the course of an hour. In this case it has lasted five days. The loss of blood has not been very great, and yet the patient says the bleeding has been continuous. The occurrence of persistent bleeding after the extraction of teeth does not always depend upon the same cause. Sometimes, after the extraction of teeth, as in fracture of the jaw, an artery of some size may be injured, but ordinarily the cause is found to be a tendency on the part of the patient to bleed whenever wounded. There is some evidence that this man is a " bleeder." He had hemorrhage from slight cause in England about eight years ago, lasting for a week. When he cuts himself the bleeding lasts for a considerable time. In some of these cases life is threatened, or even destroyed, where the bleeding occurs as the result of operative interference. I see the sockets of two teeth which are in a bleeding state. One of these is that of the left lateral incisor. The place where it has bled is marked by the presence of a plug of coagulum. There has also been bleeding from the sockets of the first or second molar.

*Treatment.*—I should suggest plugging of the sockets of the teeth with a piece of cotton impregnated with perchloride or persulphate of iron. If this fails to stop the bleeding, I should recommend the application of actual cautery. A case of this sort recalls to mind the fact that persons in whom this tendency exists should not be subjected to surgical operations. I read recently of a death which took place in a bleeder in consequence of a slight cutting operation. There are instances in which, although death has not occurred, obstinate hemorrhage has taken place, lasting a long time and nearly exhausting the patient. The hemorrhage depends upon the extreme tenuity of the blood vessels, in consequence of which they are deprived partly or wholly of their contractile power, upon the presence of which, to some extent, the arrest of bleeding depends.—*Henry B. Stubs, in Western Med. Reporter.*

---

*Treatment of Diptheria.*—Dr. J. J. O'Dea. of Stapleton, N. Y., recommends the following and, as he claims, successful local treatment of Diptheria: To the entire inflamed surface surrounding the false membrane, and close up to its border, he applies, by means of a cotton-wad, the following solution:

℞. Argenti nitrat, cryst......,............................℈ j

Spt. æther, nit. dulc....................................iv,

Aquæ destill.........  ....................................℥ iv. M.

In the same manner he then makes an application of the following mixture to the surface of the false membrane. and out to its extreme edge, but no farther:

℞. Acid. carbol. ....................................gr. viij.

Liq. ferri subsuph..................................℥ ijss,

Acid. sulphurosi...................................℥ iijss.

Glycerine............................................℥ j.

M

These are to be repeated twice, or possibly three times, in twenty-four hours, the second mixture to be supplemented by a gargle of lime water, thus allaying irritation and removing the *debris* of false membrane broken down by the action of the acid. When nothing remains of the deposit save some milky white patches he omits the applications and employs only the lime-water gargle or spray.—*Med. Record.*

